# 

**Published:** 1847-04

**Authors:** 


### BOOKS RECEIVED FOR REVIEW.

1. Remarks on Medical Organization and Reform. By E. Lee, Surgeon. Lond. 1846, pp. 121.

2. Transactions of the Medical and Physical Society of Bombay, for the Year 1844. No. VII. Bombay, 1844. 8vo, pp. 206.

3. Het Speeksel uit een Physiologisch, Diagnostisch en Therapeutisch oogpunt beschouwd. Door S. Wright, M.D. Naar de Koogduitsche Bearbeiding. Van Dr. S. Eckstein. Amersfoort, 1846. 8vo, pp. 220.

4. Memoria sobre el Cuerpo de Salud Militar, seguida de un Projecto de Reglamento del Mismo. Por Pedro Vanderlinden, M.D. Mexico, 1845. 8vo, pp. 48.

5. Reglamento del Cuerpo Medico-militar, espedido en virtud del decreto de 12 de Febrero, 1846. Mesuco, 1846.

6. Der Vaccinprocess und seine Crisen. Von A. F. Zöhrer. Wien, 1846. 8vo, pp. 213.

7. Pathologische Studien. Door Dr. C. Gobée. Eerste Deel, 1843. Tweede Deel, 1844. Utrecht. 8vo, pp. 237, 279.

8. Opere di Maurizio Bufalini, Professore della Clinica Medica. Vol. I. Parte I, II. Firenze, 1844. 8vo, pp. 337, 1846.

9. Elementi di Pathologia umana di Luigi Bosi. Tomo primo, 1843. Tomo secundo, 1844. Ferrara, 1843-4. 8vo, pp. 449-516.

10. Nuovi Elementi di Medicini eclettica del Dott. Niccolo Celle. Pisa, 1841. 8vo, pp. 487.

11. Homœopathy viewed in connexion with Medical Reform. By H. R. Madden, M.D. Lond. 1846. 8vo, pp. 168.

12. Chemistry and Physics in relation to Physiology and Pathology. By Baron Liebig, M.D. F.R.S. London, 1846. 8vo, pp. 116.

13. Guy's Hospital Reports. Second Series. Vol. IV. London, 1846. 8vo, p. 498.

14. A Guide for the proper Treatment of the Teeth. By W. K. Bridgman, Dentist. London, 1846. 12mo, p. 88.

15. The Moral Aspects of Medical Life, consisting of the 'Akesios' of Professor Marx; with Biographical Notices, &c. By James Mackness, M.D. London, 1846. 8vo, pp. 348.

16. Chemistry of the Four Seasons. By T. Griffiths. London, 1846. 8vo, pp. 495.

17. Practical Observations and Suggestions in Medicine. Second Series. By Marshall Hall, M.D. London, 1846. 8vo, pp. 360.

18. Practical Remarks on Near Sight, Aged Sight, and Impaired Vision. By W. W. Cooper, Surgeon. London, 1847. 8vo, pp. 216.

19. Observations on Hydropathy; with an Account of the principal Cold-Water Establishments in Germany. By J. S. Buchnan, M.D. Berlin and London, 1846. 8vo, pp. 170.

20. Liebig's Question to Mulder tested by Morality and Science. By Dr. G. T. Mulder, Professor of Chemistry in the University of Utrecht. Translated by Dr. P. T. H. Fromberg. Edinburgh, 1846. 8vo, pp. 124.

21. The Motives to Industry on the Study of Medicine; an Address delivered at St. Bartholomew's Hospital. By James Paget, F.R.C.S. Lond. 1846. 8vo. pp. 30.

22. The Potato Plant, its Uses and Properties; together with the cause of the present Malady. By A. Smee, F.R.S. London, 1846. 8vo, pp. 174.

23. Animal Chemistry, or Chemistry in its Applications to Physiology and Pathology. By Baron Liebig. Edited from the Author's MS., by W. Gregory, M.D. Third edition. Part I. Lond. 1846. 8vo, pp. 253.

24. The Stars and the Earth; or Thoughts upon Space, Time, and Eternity. London, 1846. 12mo, pp. 48.

25. On the Correlation of Physical Forces. By W. R. Grove, Esq., M.A. F.R.S. London, 1846. 8vo, pp. 52.

26. On Medical Education; a Lecture delivered at King's College. By W. A. Guy, M.B. London, 1846. 8vo, pp. 64.

27. Lectures and Observations on Clinical Surgery. By And. Ellis. Dublin, 1846. 8vo, pp. 275.

28. Lectures on Clinical Medicine. Diseases of the Heart. By P. M. Latham, M.D. Vol. II. London, 1846. 8vo.

29. Elements of Chemistry. By Thos. Graham, F.R.S. Second edition, entirely revised. Part I. London, Nov. 1846. 8vo, pp. 160.

30. The Pathological Anatomy of the Human Body. By Julius Vogel, M.D. Translated by G. E. Day, M.A. L.M. Cantab. London, 1847. 8vo, pp. 585. With ten plates.

30. An Essay on the Tongue, in Functional Derangement of the Stomach and Bowels, and on the appropriate Treatment. By E. Williams, M.D., Physician to the Essex and Colchester Hospital. Second edition, carefully rewritten, with much additional matter. London, 1846. 8vo, pp. 236.

31. Handbook of Human Anatomy, general, special, and topographical. Translated from the German of Dr. A. Von Behr, and adapted to the use of the English Student. By John Birkett, Surgeon, Demonstrator of Anatomy at Guy's Hospital. London, 1846. 8vo, pp. 457.

32. The Climate of Torquay. By E. Vivian, Esq. London, 1846. 8vo.

33. A Manual of Materia Medica and Therapeutics. By J. F. Royle, M.D. F.R.S. London, 1847. 8vo, pp. 171.

34. Principles of Human Physiology. By W. B. Carpenter, M.D. F.R.S. Third edition. Lond. 1846. 8vo, pp. 776.

35. Knowledge; an Introductory Address delivered at the Bristol Athenæum, Oct. 5, 1846. By J. A. Symonds, M.D. Lond. 1846. 8vo, pp. 47.

36. A Manual of the Principles and Practice of Ophthalmic Medicine and Surgery. By T. Wharton Jones, F.R.S. London, 1847. 8vo, pp. 570.

37. Hygiea. Medicinsk och Pharmaceutisk

Mändas-Skrift. Redactörer: Dr. Carlson, Sonden-Berg, Huss, Retzius. Bandet 1844-5-6. Stockholm.

38. Svenska Läkare-Sållskapetts nya Handlingar. Bandet I, II, III, IV, V. Stockholm, 1837-8; 1843-4 5. 8vo.

39 Om Bright'ska Njursjukdomen. Akademisk Afhandling Af Pehr Henrik Malmsten, M.D, Stockholm, 1842. 8vo, pp. 120.

40. Ueber die Bright'sche Nierenkrankheit. Von Peter H. Malmsten. Uebersetzt von G. Von dem Busch, M.D. Bremen, 1846.

41. On Diseases of the Skin. By Erasmus Wilson, F.R.S. Second edition; with eight coloured plates London, 1847. 8vo, pp. 482.

42. Homöopathie, Allopathie, und die neue Schule. Von John Forbes, M.D. Bearbeilet Von Adolph Bawr, M.D. Wien, 1846. 8vo, pp. 70.

43. A Treatise on the Plague; more especially on the Police Management of that Disease. By A. White, M.D., Deputy Inspector-General of Military Hospitals. London, 1847. 8vo, pp. 342.

44. The India Journal of Medical and Physical Science, for 1845. Twelve numbers. Edited by C. Finch, M.D. Calcutta, 1845.

45. Clinical Facts and Reflections; also Remarks on the Impunity of Murder in some Cases of presumed Insanity. By Thomas Mayo, M.D. F.R.S. London, 1847. 8vo, pp. 217.

46. A descriptive Catalogue of the Anatomical Museum of St. Bartholomew's Hospital. Vol. I. Pathological Anatomy. Lond. 1846. 8vo, pp. 487.

47. Urinary Deposits; their Diagnosis, Pathology, and Therapeutical Indications. Second Edition. By Golding Bird, M.D. London, 1846. 8vo, pp. 356.

48. Chemistry and Physics, in relation to Physiology and Pathology. By Baron Liebig. London, 1846. 8vo, pp. 116.

49. Dr. Underwood's Treatise on the Diseases of Children; with Directions for the Management of Infants. Tenth edition, with additions. By Henry Davies, M.D. Lond. 1846. 8vo, pp. 595.

50. The Microscopic Anatomy of the Human Body. By A. H Hassall. Part III. Oct. 1846.

51. On the Pathology and Treament of Scrofula; being the Fothergillian Prize Essay for 1846. By R. M. Glover, M.D., Lecturer in the Newcastle Medical School. London, 1844. 8vo, pp. 315.

52. Researches into the Physical History of Mankind. By J. C. Prichard, M.D. F.R.S., &c. Vol. 5, concluding the work. London, 1847. 8vo, pp. 570.

53. Lectures on the Comparative Anatomy and Physiology of the Vertebrate Animals. By Richard Owen, F.R.S., &c. Part I. London, 1846. 8vo, pp. 808.

54. Elements of Chemistry. By the late Edward Turner, M.D., &c. Eighth edition. Edited by Baron Liebig and W. Gregory, M.D., &c. Part I. Inorganic Chemistry. London, 1847. 8vo, pp. 676.

55. Practical Observations on some of the Diseases of the Stomach and Alimentary Canal. By James Alderson, M.D. F.R.S. London, 1847. 8vo, pp. 215.

56. Body and Soul, or Life, Mind, and Matter, &c. By G. Redford, Surgeon. London, 1847. 8vo, pp. 232.

57. Dictionary of Practical Medicine. Part XI. By J. Copland, M.D.

58. The Nature and Faculties of the Sympathetic Nerve. By Joseph Swan. London, 1847. 8vo, pp. 55.

59. An Inquiry into the Action of Mercury on the Living Body. By Joseph Swan. Third edition. London, 1847. 8vo. pp. 34.

60. On Indigestion, and certain Bilious Disorders often connected with it. To which are added short Notes on Diet. By G. C. Child, M.D. Lond. 1847. 8vo, pp. 219.

61. Observations on Aneurism, and its Treatment by Compression. By O'Bryan Bellingham, M.D. &c. London, 1847. 8vo. pp. 181.

62. On Tumours of the Uterus and its Appendages. (Jacksonian Prize Dissertation.) By T. S. Lee, Surgeon. London, 1847. 8vo, pp. 274.

63. A Treatise on the Inhalation of the Vapour of Ether, for the prevention of Pain in Surgical Operations, &c. By J. Robinson, Surgeon-Dentist. London, 1847. 8vo.

64. The London and Provincial Medical Directory, 1847. London, 8vo, pp. 862.

65. Andeutungen über Ventilation. Eine Anrede an gebildete Nutaerzte. Von E. Sieveking, M.D. Homburg, 1846. 8vo, pp. 69.

66. Proceedings of the Lincoln Lunatic Asylum; and Communications with her Majesty's Commissioners in Lunacy. London, 1847. 8vo, pp. 97.

67. Observations on the History and Treatment of Dysentery and its Combinations, &c. &c. By W. Hartz, M.D. Second edition. Dublin, 1847. 8vo, pp. 303.

68. On the Mechanism of Respiration. By F. Sibren. From the Philosophical Transactions for 1846. 4to.

69. On Cataract, Artificial Pupil, and Strabismus. By F. H. Brett, Esq. M.D. London, 1847 8vo, pp 89.

70. Medical Statistics; their Force and Fallacies. A Lecture. By J. F. Duncan, A.M. M.B. Dublin, 1847. 8vo, pp. 42.

71. Outlines of Structural and Physiological Botany. By A. Henfrey, F.L.S. London, 1847. 8vo, pp. 234.

72. A Lecture on the Sanitary Condition of Newcastle-on-Tyne, and the Means necessary for its Improvement. By George Robinson, M.D., &c. Newcastle-on-Tyne, 1847. 8vo, pp. 58.

73. A Treatise on Diet and Regimen. By W. H. Robertson, M.D. Part I.

74. Gray's Supplement to the Pharmacopœia. Rewritten and enlarged by Thomas Redwood. London, 1847. 8vo, pp. 1118.

### INDEX TO VOL. XXIII

OF THE

### BRITISH AND FOREIGN MEDICAL REVIEW.

PAGE

Abscess, hepatic, in India . . 149
Adams, Dr. his Paulus Ægineta . 251
Addison, Dr. on phthisis . . 421
Affections, inflammatory . . 302
Ague, in early childhood . . 305
Alterations, pathological . . 55
Amenorrhœa, cases of . . . 291
Anatomy, microscopic, Mr. Hassall on 252
    textural, Prof. Burggraeve on 136
    pathological, Dr. Gross's elements of . . . 64
Angionosi . . . . . . 56
Animals, Boussingault on the food of 157
    Liebig on the food of . 157
    Dr. Thomson on the food of 157
Arachnitis, cerebro-spinal, epidemic 302
Artificial anus, Dr. Dieffenbach on 1
    treatment of . . 27
Ashwell, Dr. on diseases of women 239, 612
Asphyxia neonatorum . . . 300
Balfour, Dr. on the Vienna School and on homœopathy . 607
Baly, Dr. on mortality in prisons . 411
Barker, Dr. on aneurism . . 416
Barlow, Dr. clinical reports . . 419
Behr's handbook of human anatomy, by Birkett . . . . . . 255
Bengal medical returns . . . 440
Bird, Dr. G. on urinary deposits . 248
    diseases of children . 424
Body and Soul, Mr. Redford on . 576
Blood, buffy coat of . . . 507
Body, morbid habits of . . . 59
Bone, Dr. on the treatment of yellow fever . . . . . . 426
Books received for review . . 611
Boussingault on the food of animals 157
Boyd, Dr. on malformation of the duodenum . . . . . . 412
Brereton, Mr. medical reports . . 421
Bright's disease, urine in . . 504
Brittan, his edition of Malgaigne's operative surgery . . . 155
Brodie, Sir B. on medical education 242
Burggraeve, Dr. his textural anatomy . . . . . . 136
Bigelow, Dr. on ether . . . 310
Cæsarean section, cases of . . 284

PAGE

Carpenter, Dr. on human physiology 245
Cephalhæmatoma, Dr. West on . 413
Chemistry, Mr. Griffiths on . . 238
Chemistry, Dr. Turner's . . . 584
Child, Dr. on indigestion . . 577
Childhood, diseases of . . 37, 302
Cholera, Asiatic, Drs. Copland, Kennedy, and Lorimer on . . 313
Clark, Mr. Le Gros, on the urethra 414
Cleft palate, its treatment . . 411
Combe, Mr. on national education . 254
    Dr. on the observation of nature in the study and treatment of disease . . 257
Contagion, Dr. Holland on . . 313
Convulsions, puerperal . . . 289
Cooper, Mr. Bransby, on fracture of the thigh-bone . . . . 425
Cooper, Mr. on sight . . . 511
Copland, Dr. on Asiatic cholera . 313
Craniotomy, case of . . . 284
Curling, Mr. case of remarkable hypertrophy . . . . 413
Davies, Dr. on diseases of children 237
Davy, Dr. on the colostrum of the cow . . . . . . 411
Day, Dr. his Vogel's anatomy . 280
Delirium tremens, Dr. Ware on . 603
Degenerations . . . . . 57
Derangement, mental, Dr. Moreau on 217
Diarrhœa in childhood . . . 304
Dieffenbach, his operative surgery . 1
    treatment of artificial anus 27
Diet, in infancy . . . . 299
Digestion, diseases of the organs of 304
Digitalis, on the action of . . . 417
Diseases, acute inflammatory . . 61
    distribution of into classes 61
    radical . . . . 61
    transitory contagious . 61
    of India, Dr. Geddes on . 106
    of the heart . . . 169
    of children . . . 237
    of women . . . 239
    natural history and treatment of . . . . 257
    uterine appendages . . 295
Dissolvents . . . . . 49

PAGE

Dyscrasiæ, &c. . . . . . 308
  evacuations in . . 504
  complications . . 150
  causes of . . . 150
Dysentery in India . . . 147
Dysmenorrhœa, valerianate of zinc in 292
Dixon, Mr. on anæsthenia . . 413
Education, national, Mr. Combe on 254
Ellis, Mr. on clinical surgery . . 250
Emiliani, Prof. on medical history . 86
Entozoa, sarconotic . . . 58
Ether, use of in surgical operations . . . . . 309
  the discovery of . . ib.
  Mr. Robinson on . . 547
  Prof. Simpson on . . ib.
  M. Longet on . . . 570
  M. Flourens on . . 572
  Dr. Snow on . . . 579
Evans, Mr. on obstruction of the large intestine . . . . 411
Fergusson, Mr. on cleft palate . 411
  on morbus coxarius . 415
Fever, Prof. Lanza's views of . . 56
  Dr. Reed on . . . 241
  Mr. Stallard on the treatment of 269
  treatment of by cold water . ib.
  puerperal . . . . . 290
  typhoid, in Prague . . 306
  yellow, treatment of . . 426
Fevers . . . . . . . 305
  acute continued . . 61
  periodic endemic . . ib.
Fistula, vesico-vaginal . . . 297
Fœtation, extra-uterine . . . 425
Fœtus, diseases of . . . 298
Food of animals. . . 157, 492
Fractures, Dr. Walker on . . 256
France, Mr. on iritis . . . 418
  crystalline lens . . 425
Francis, Dr. on sudden death from disease . . . . . . 424
French military memoirs . . 74
Fungi, in the mouth . . . 509
Gangrene, infantile . . . 308
Geddes, Dr. on diseases of India . 106
Gellerstedt, Dr., on tubercular phthisis 429
Gely, Dr. on wounds of the intestines 1
Glanders, Remak on . . . 507
Glover, Dr. on scrofula . . . 513
Greatrex, Mr. on aneurism . . 410
Gregory, his Turner's chemistry . 584
  his pathological anatomy . 64
Griffith, Dr. on the blood and secretions . . . . . . 254
Griffiths, Mr. his chemistry of the four seasons . . . . . 238
Gross, Dr. on wounds of the intestines . . . . . . 1

PAGE

Gulliver, Mr. his edition of Hewson 251
Guy, Dr. on the health of towns . 240
  on medical education . 242
Guy's hospital reports . . . 416
Hachisch, Dr. Moreau on . . 217
Hall, Dr. M. his observations in medicine . . . 185
  his views anticipated by Prochaska and others . . . 202
Harvey, records of, by Mr. Paget . 249
Hassall, Mr. on microscopic anatomy 252
Head, diseases of . . . . 119
Health of towns, Dr. Guy on . . 240
Heart, Dr. Latham on the diseases of 169
Hemorrhage, uterine . . . 284
Hepatitis in India . . . . 152
  causes of . . ib.
Hessler, Dr. his edition of Susruta . 521
Hernia, moveable, treatment of . 34
Hewett, Mr. on extravasations . 410
Hippocrates' works, by M. Littré . 253
Holland, Dr. on contagion . . 313
Hughes, Dr. on paracentesis . 419
Hydrocephalus, acute, symptoms of 302
  chronic . . 303
Hygiene, military . . . . 440
Imperforate anus, curious case of . 301
India, on the dysentery of . . 146
Indigestion, Dr. Child on . . 875
Infancy, diet in . . . . 299
  diseases of . . . 300
Infiltration, tubercular . . . 431
Inflammation, abdominal . . 126
  hepatic . . . 120
  thoracic . . . ib.
  and ulceration of the os and cervix uteri 293
Intestines, wounds of . . . 1
  nature of wounds of . 2
  treatment of wounds of . 6
  wounds of, cautery in . 31
  running suture in 32
Irritants . . . . . . 49
Iritis, remarks on . . . 418
Jones, Mr. Wharton, on ophthalmic medicine . . . . . 246
Kennedy, Dr. on epidemic cholera. 313
King, Mr. on fracture of thigh-bone 419
Knowledge, Dr. Symonds on . . 252
Labour, natural . . . . 279
  preternatural . . . 280
Lanza, Dr. his positive nosology . 37
Larynx, ulcers in the . . . 431
Latham, Dr. on diseases of the heart 169
Lectures, Dr. Latham's clinical . ib.
Lee, Dr. on puerperal ophthalmia . 413
Lesions . . . . . . 56
Lever, Dr. on protracted labour . 424

PAGE

Leupoldt, Dr. his characteristics of medicine . . . . . . 86

Liebig, on the food of animals . 157

Liebig's animal chemistry . . 492

Liebig, his Turner's chemistry . 584

Littré, M. his works of Hippocrates 253

Liver, abscess of . . . . . 123

Lorimer, Dr. his report on the epidemic cholera . . . . . 313

Lungs, caverns in the . . . 431

Mackness, Dr. his moral aspects . 467

Malgaigne's operative surgery, by Dr. Brittan . . . . . . . 152

Marshall, Mr. on military punishments . . . . . . 163

Materia Medica, Dr. Royle on . 244

Marx, Prof. his tribute to Stieglitz . 544

Matteucci's lectures on physical phenomena . . . . . 377

Measles, epidemic of . . . . 306

Medical education, Sir B. Brodie on 242

Dr. Guy on . ib.

Mr. Paget on . ib.

Medical life, moral aspects of . 467

police of the United Kingdom 86

Medicine, positive, Dr. Lanza on . 37

philosophy of . . 39

its past, present, and future 86

ancient Indian . . 521

Dr. M. Hall's observations in . . . . . 185

Medico-chirurgical transactions . 410

Memoirs on military medicine, &c. 74

Menorrhagia, oxide of silver in . 292

Menstruation, disorders of . . 291

Midwifery, report on . . . 277

operative . . . 283

Military medicine, memoirs on . 74

Morbid anatomy, neglect of by examining corporations . . . 65

Moreau, Dr. his psychological studies 217

Mouat, Dr. his observations on the Bengal medical returns . . 440

Munk, Dr. on digitalis . . . 417

Nerve, sympathetic, Mr. Swann on . 558

Neuronosi . . . . . . 55

Nosology, positive, Dr. Lanza on . 37

Nottingham, Dr. on popliteal aneurism . . . . . . . 425

Oldham, Dr. on extra-uterine fœtation . . . . . . ib.

Ophthalmic medicine, Mr. Wharton Jones on . . . . . . 246

Opium, case of poisoning by . . 416

Ovaria, diseases of the . . . 295

Owen, Prof. his lectures . . 472

Paget, Mr. on medical education . 243

his records of Harvey . 249

on obstructions of arteries 413

PAGE.
Parkes, Dr. on the dysentery and hepatitis of India . . . 146
Pathology, clinical . . . 47
Peacock, Dr. on obstruction of the vena cava . . . . . 410
Pelvis, malformation of the . . 280
Pericarditis, causes of . . . 415
Peritonitis in new-born children . 301
Philosophy of medicine . . . 39
Phthisis, pathology of . . . 421
pneumonic . . . ib.
tuberculo-pneumonic . ib.
tubercular . . . 423
Dr. Gellerstedt on 429
Phthisis, table of seasonal influence on 436
causes of . . . 437
treatment of . . . ib.
Phymosis, surgical treatment of . 155
Physical forces, Mr. Grove on . 247
Physicians, the periodeutic . . 91
Physiology, human, Dr. Carpenter on 245
Plurality of birth . . . . 280
Pneumonia, expectoration in . . 504
Polypus uteri . . . . 293
Porrigo lupinosa . . . . 508
Potato, the, Mr. Smee on . . 751
Pregnancy, disorders of . . . 277
signs of . . . ib.
case of fallopian . . 278
extra-uterine . . ib.
Prochaska, his anticipation of Dr. M. Hall's views . . . 202
on the functions of the common sensory . 20[illegible]
Pseudo-organic productions . . 58
Punishment, military, Mr. Marshall on 163
Rainey, Mr. on the lungs . . 415
Reed, Dr. on fever . . . . 241
Remak, Dr. his investigations . 503
Renouard, his history of medicine . 86
Report on midwifery, by Dr. West . 277
Reports, select clinical . . . 419
Respiration, diseases of the organs of 303
Rheumatism in the Indian army . 126
Royle, Dr. his materia medica . 244
Saline medicines, in yellow fever . 426
Saliva, Dr. Wright on . . . 129
Scarlatina, its sequelæ . . . 306
urine in . . . 504
Schönlein's investigations, by Dr. Remak . . . . . 503
Scrofula, Mr. Phillips on . . 308
Dr. Glover on . . 513
state of blood in . . 517
Scrofulous products . . . 515
Sight, Mr. Cooper on . . . 511
Skin diseases, Mr. E. Wilson on . 251
Simpson Prof. on ether . . . 547
Spermatorrhœa . . . . 508

PAGE
Spina bifida, treatment of . . 300
Sri Madusa dana Gupta, his edition of Susruta . . . . . 521
St. Bartholomew's hospital, catalogue of the museum of . . . 249
Stallard, Mr. on fever . . . 269
Stanley, Mr. on pulsating tumours of bone . . . . . . 412
Statistics of midwifery . . . 291
Stieglitz, Prof. Marx's tribute to . 544
Surgery, clinical, Mr. Ellis's lectures on 250
Susruta on ancient Indian medicine 521
Swaine, Dr. his translation of Hasse on diseases of the respiratory organs 251
Sydenham society, works published by, in 1846 . . . . ib.
Symonds, Dr. on knowledge . . 252
Systems, nosological origin of . 97
Taylor, Dr. on pericarditis . . 415
Mr. on poisoning by opium 416
medical jurisprudence 423
poisoning by arsenic . 425
Teevan, Mr. on a black secretion from the skin . . . . . 416
Theories, medical, origin of . . 97
Therapeutics, infantile . . . 299
Thomson, Dr. on the food of animals . . . . . 157, 492
Trismus, Dr. Sims's theory of. . 300
Tubercle, crude . . . . . 430
miliary . . . . . ib.
softening of . . . 434
Typhus, abdominal . . . 503.

PAGE
Underwood, Dr. on diseases of children . . . . . . 237
Urinary deposits, Dr. G. Bird on . 248
Uterus, morbid states of . . 281
diseases of . . . 292
displacements of the . . ib.
malignant diseases of . . 295
Vagina, diseases of . . . 297
Variola, anatomy and treatment of . 307
Veins, entrance of air into the . 443
table of cases of 452
Vertebrate animals, Prof. Owen on . 472
Vis medicatrix, illustrations of . 265
Vogel's anatomy, by Dr. Day . . 250
Walker, Dr. on fractures . . 256
Ware, Dr. his letter on ether . 309
on delirium tremens. . 603
Why and Wherefore, the . . 759
Warren, Dr. his note on ether . 344
Wattman, Dr. on air in veins . 724
Webster, Dr. on mental diseases . 174
West, Dr. his report on midwifery, &c. ib.
on cephalhæmatoma . 413
Wise, Dr. on the Hindu system of medicine . . . . . . 521
Wright, Dr. on saliva . . . 129
Wounds of the intestines, Dr. Gross on 1
table of gunshot . . 79
Women, diseases peculiar to . . 62
Dr. Ashwell on diseases of 239
Wilson, Mr. E. on diseases of the skin 251
on the echinococcus hominis . . 410

END OF VOL. XXIII.

C. AND J. ADLARD, PRINTERS, BARTHOLOMEW CLOSE

